# nuTCRacker: Predicting the Recognition of HLA‐I–Peptide Complexes by αβTCRs for Unseen Peptides

**DOI:** 10.1002/eji.202451607

**Published:** 2025-07-09

**Authors:** Justin Barton, Trupti Gore, Meghna Phanichkrivalkosil, Adrian Shepherd, Michele Mishto

**Affiliations:** ^1^ School of Natural Sciences and Institute of Structural and Molecular Biology Birkbeck University of London London UK; ^2^ Centre for Inflammation Biology and Cancer Immunology & Peter Gorer Department of Immunobiology King's College London London UK; ^3^ Francis Crick Institute London UK

**Keywords:** antigen presentation/processing, bioinformatics, MHC, TCR

## Abstract

The ability to predict which antigenic peptide(s) the αβTCR of a given CD8^+^ T‐cell clone can recognise would represent a quantum leap in the understanding of T‐cell repertoire selection and development of targeted cell‐mediated immunotherapies. Current methods fail to make accurate predictions for antigenic peptides not present in the training dataset. Here, we propose a novel deep learning method called nuTCRacker that makes accurate predictions for a subset of unseen peptides, with an AUC > 0.7 for around a third of peptides evaluated using a large dataset compiled from curated public resources. An additional evaluation was undertaken using a small cellula‐validated dataset of αβTCR peptides associated with cancer. Our analysis suggests that it is possible to make useful predictions for an unseen peptide provided the training dataset contains: many samples with the same HLA class I molecule as that bound to the peptide; at least one peptide that is similar to the target peptide; and a small number of αβTCRs that are similar to those bound to the unseen peptide of interest.

## Graphical abstract

nuTCRacker aims to predict unseen αβTCR‐peptide‐HLA‐I affinity. Predictions are successful for only a subset of unseen peptides although they are better than the benchmarked methods. Predictions are tested on both novel and published T cell assays.

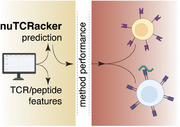

## 1 Introduction

The human leucocyte antigen class I (HLA‐I) antigen presentation pathway, whereby peptides are presented for inspection by αβT cell receptors αβTCRs present on the surface of CD8^+^ T cells, is one of the core elements of the adaptive immune system and plays a crucial role in fighting intracellular pathogens and in the eradication of tumour cells [1]. In addition, the avidity of αβTCRs on thymocytes—the precursors of mature CD8^+^ T cells in the periphery—for self‐antigenic peptides presented by HLA‐I molecules in the cortex and medulla of human thymi is considered a key factor in the positive and negative selection processes that underpin central tolerance [2].

Advances in high throughput sequencing techniques have led to an explosion in the number of known αβTCR sequences, with resources such as the iReceptor [3] providing public access to billions of TCR β chains and increasing numbers of paired TCR α and β chains. At the same time, the number of peptides known to be presented by HLA‐I molecules is increasing rapidly, partly owing to the development of high‐throughput elution‐based strategies and increasingly precise bioinformatic strategies for the correct identification of both canonical and noncanonical peptides bound to HLA‐I molecules [4, 5, 6, 7, 8, 9, 10]. Moreover, computational methods for predicting peptide‐HLA‐I binding are more and more efficient and widely used and have become the core of most of the pipelines predicting novel epitope candidates and optimizing vaccine development [11, 12, 13, 14].

There remains, however, a critical gap to link the specific recognition by CD8^+^ T cells to what is presented by HLA‐I molecules and the degrees of cross‐recognition of multiple peptide‐HLA‐I: in all but a tiny fraction of cases, we don't know what peptide‐HLA‐I complexes a given αβTCR can bind to with sufficient affinity to facilitate T cell activation in the periphery or with the right affinity to pass central tolerance selection in the thymus. This knowledge is essential for understanding the basic nature of CD8^+^ T cell repertoire formation, and supports cancer immunotherapy design by aiding the identification of TCRs that target patient‐specific neoantigens, although CD8^+^ T cell activation goes beyond the complex αβTCR recognition of peptide‐HLA‐I complexes [1, 15, 16]. There are in silico—in cellula methods for the identification of peptide‐HLA‐I sequences recognised by orphan αβTCRs, although they do require a strong immunological background and skills for laboratories that would like to take that path [17]. Having reliable models able to predict the specific binding of a given αβTCR to peptide‐HLA‐I complexes would act as a driving force for research in many medical fields. However, from a computational perspective, predicting the αβTCR‐peptide‐HLA‐I binding affinity has proved much less tractable than the peptide‐HLA‐I binding affinity prediction, the latter being tightly constrained (in terms of peptide conformation and the presence of distinct binding pockets that accommodate specific peptide sidechains), such that a moderate degree of accuracy is possible even with simple matrix‐based approaches. Contrastingly, the αβTCR‐peptide‐HLA‐I binding involves at least a subset of the αβTCR's flexible complementarity determining regions (CDRs) engaging with the comparatively flat surface formed by a peptide‐HLA‐I complex.

Although some progress in αβTCR‐peptide‐HLA‐I binding prediction has been made using classical machine learning methods such as Random Forest, recent research has focused on deep learning, with numerous methods developed since 2020 that vary those in terms of the input data they utilize, how that data are encoded and the choice of deep learning architecture [18, 19] (Table S1). Robust performance comparisons based on these methods’ published levels of prediction accuracy are impossible, given that different training and evaluation data have been used in their development. There is broad acceptance that, so far, useful levels of αβTCR‐peptide‐HLA‐I binding prediction accuracy appear possible for peptides that occur within the training set, that is, predicting whether an unseen αβTCR is capable of binding to a given peptide‐HLA‐I complex that occurs within the training set bound to different TCRs. It has been estimated that as many as 50 to 200 αβTCRs bound to the same target peptide are required to make good predictions with the current model [20, 21]. In contrast, generalizing the αβTCR‐peptide‐HLA‐I binding prediction for unseen peptides (where no αβTCRs capable of binding to that peptide are present in the training set) remains intractable [22, 23, 24]. Unfortunately, the latter is exactly what we would need a predictor to do, to fully understand, for example, what antigenic peptides (if any) drive the CD8^+^ T cell repertoire selection in human thymi or what (neo)epitopes are recognised by tumour‐infiltrating lymphocytes and could cancer patients be vaccinated against.

## 2 Results

### 2.1 Dataset Preparation for Model Training

To address these issues, we developed a novel αβTCR‐peptide‐HLA‐I binding predictor (Novel Universal Transformer for Cross‐epitope Recognition using Advanced Cross‐domain Knowledge Extraction and Representation, nuTCRacker), benchmarked it with other models, and applied it to a small set of αβTCR‐peptide‐HLA‐I experimentally tested in our laboratory.

**FIGURE 1:**
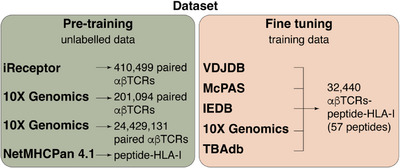
Data from five public databases used for model cross‐validation.

The first key and challenging step for model development and training is dataset preparation. To this end, we prepared two kinds of datasets (Figure 1). The first contained unlabelled data—both αβTCRs of unknown peptide specificity and peptide‐HLA‐I data with unknown cognate TCRs—whereas the second contained labelled data with αβTCRs matched to peptide‐HLA‐I complexes.

The former kind of dataset was used to explore the potential benefits of adopting transfer learning approaches in the context of αβTCR‐peptide‐HLA‐I binding prediction. Bulk αβTCR repertoire data were downloaded from iReceptor [3], selecting only productive sequences (i.e., sequences whose gene rearrangements would produce a functional receptor) from the human loci TRA or TRB with specified CDR3, and V and J genes. The CDR1 and CDR2 were not explicitly required as these are completely determined by the V gene allele. Duplicate sequences were removed, resulting in 410,499 paired (i.e., comprising both the α and β chains of a given αβTCR) and 24,429,131 unpaired (i.e., only a single α or β chain from a given αβTCR) sequences. Additional bulk αβTCR data were downloaded from 10x Genomics (10x Genomics, 2023), and filtered to include only αβTCR data with high confidence, resulting in 201,094 paired sequence records. We found that it was sufficient to pre‐train only with the paired sequence records, saving substantial pretraining compute. This is consistent with findings in antibody language models [25]. With respect to the peptide‐HLA‐I component, bulk data were downloaded from the training set provided with NetMHCpan 4.1 [26] and filtered to include only peptide‐HLA‐I complexes where the binding has been experimentally confirmed.

For the latter kind of dataset, αβTCR‐peptide‐HLA‐I binding data were downloaded from five public databases and repositories, *i.e*., VDJdb [27], McPAS‐TCR [28], TBAdb [29], IEDB [30] and 10x Genomics (10x Genomics, 2023). All peptides included in this dataset did not have post‐translational modifications (PTMs), which could alter the αβTCR‐peptide‐HLA‐I binding [1]. Downloaded data were filtered to only include human loci TRA or TRB with specified CDR3, V and J genes associated with peptides 8–12 amino acids long. Across all the preceding datasets, relevant fields were converted to a single, standardised format, all records were combined in a single set, and duplicates were removed. Negative data were created by simulating non‐binding αβTCR‐peptide‐HLA‐I pairs via the random mispairing of αβTCRs and peptide‐HLA‐I from this aggregated dataset, a strategy widely adopted in other studies. Given that half of the positive samples were associated with a single cytomegalovirus (CMV) epitope, these were down‐sampled to give approximately the same number as that associated with the second most frequent epitope, giving a total of 2200 positive samples. An equal number of negative samples was generated, giving 32,440 αβTCR‐peptide‐HLA‐I samples in our dataset (Figure 1). This 50/50 ratio of negatives to positives was preferred to a higher proportion of negatives because our chosen methodology proved insensitive to this ratio in preliminary tests, training times were shorter, and generating a large number of additional negative samples would entail additional down sampling of positive samples associated with epitopes that were highly represented.

Since our approaches required access to either full αβTCR amino acid sequences or to all their CDR subsequences—whereas many TCR binding data repositories stored only a minimal description of a given αβTCR consisting of its CDR3 sequences and its V and J gene identifiers—Stitchr [31] was used, in the latter cases, to reconstitute the complete α and β chain amino acid sequences from their minimal descriptions.

In the case of HLA‐I molecules, we explored the use of pseudosequences as a concise alternative to lengthy HLA‐I amino acid sequences. We used an HLA‐I pseudo‐sequence that comprised the set of 34 amino acid residues specified by Nielsen and colleagues [32] based on their analysis of residues lying within 4.0 Å of bound in 9 amino acid long peptides for a representative set of HLA‐A and HLA‐B structural complexes. It is worth noting that Liu and colleagues [33] used HLA‐I pseudosequences comprising 40 positions (i.e., an additional six positions compared with the pseudosequences used here). In future work, we would be interested to explore whether these improve performance.

Further details of the dataset preparation are provided in Section 4. The resulting dataset is freely accessible online (see data availability section).

### 2.2 Development of nuTCRacker

Transformer architectures have emerged as powerful tools in various domains, including natural language processing and bioinformatics. They rely on multiheaded self‐attention mechanisms that dynamically weigh input contributions according to their relevance across different positions. By doing so, transformers can effectively capture long‐range interdependencies absent in recurrent neural networks and convolutional counterparts.

To develop nuTCRacker, we employed the DeBERTa [34] architecture for predicting the αβTCR‐peptide‐HLA‐I binding based on the aforementioned amino acid sequence inputs. DeBERTa improves on the successful BERT foundation with a disambiguated self‐attention mechanism. Our choice was motivated by the need to accurately capture complex and variable long‐range dependencies in the input sequences.

We trained DeBERTa models using a masked language modelling task as has proven successful in other protein language models [35, 36]. In preliminary assessments, various data configurations were evaluated, for example, utilizing different subsections of the full αβTCR sequence (for a full list of alternative strategies, see Section 4).

**FIGURE 2:**
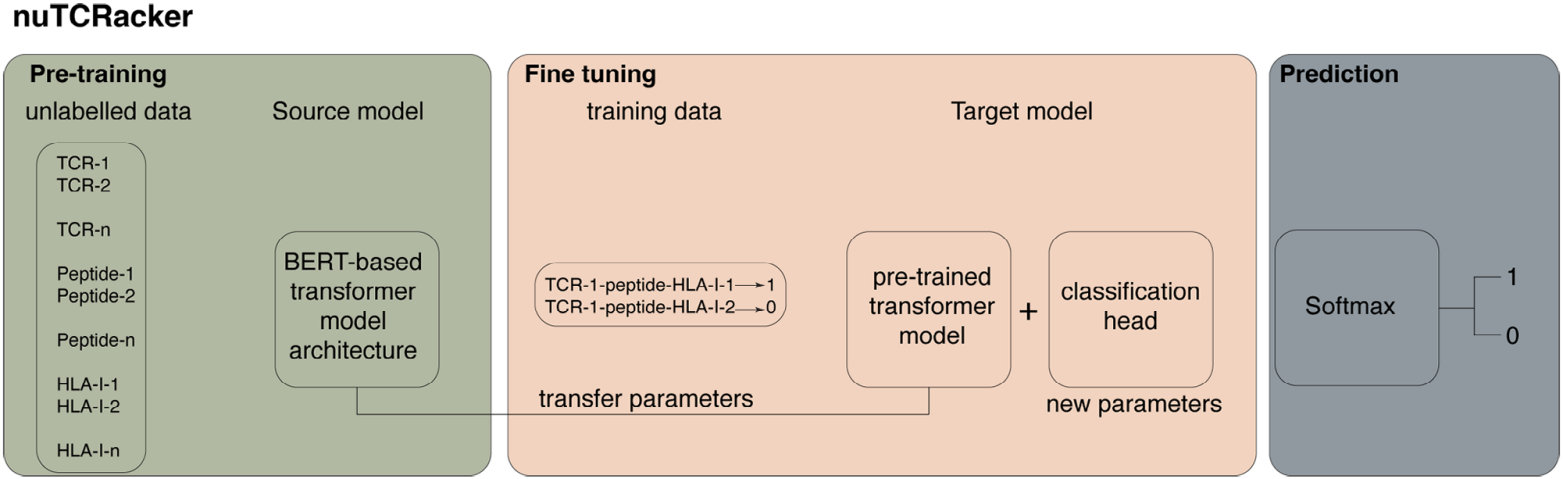
nuTCRacker. A transformer model, based on the DeBERTa architecture, was trained using a masked language modelling task using unlabelled αβTCR, peptide and HLA‐I sequences. This pretrained model was then finetuned to address the αβTCR‐peptide‐HLA‐I binding prediction task using labelled data from the databases shown in Figure 1.

Based on these preliminary assessments, a single model—nuTCRacker—was selected that took as input: the αTCR, βTCR, peptide, and HLA‐I amino acid sequences, treating each residue as a token and using prefix tokens [tra], [trb], [peptide] and [mhc], respectively, as prefixes to delimit the input components (Figure 2).

Hyperparameter optimization was performed using Bayesian optimization without reference to any of the test data. Specifically, an iterative tree‐structured Parzen estimator (TPE) model [37] was used as implemented in Optuna. TPE worked by dividing the hyperparameter space into two regions: one that contained the best‐observed values and one that contained the rest. TPE then fitted a Parzen window to each region with which it estimated the expected improvement of each proposed hyperparameter value. TPE then evaluated the value that maximized the expected improvement. TPE updated the Parzen windows and expected improvement after each iteration (Figure 2).

### 2.3 Benchmarking of Predictive Accuracy and Performance of nuTCRacker and Competing Models

To evaluate the performance of nuTCRacker, comparisons were made with five existing models: ERGO‐II [38], NetTCR‐2.1 [21, 39], pMTnet [40], ATM‐TCR [41] and TEINet [42]. Two versions of NetTCR‐2.1 were evaluated, one that took as input paired αβ chain CDR3 sequences and the other taking only the CDR3β sequence (Table S1).

**FIGURE 3:**
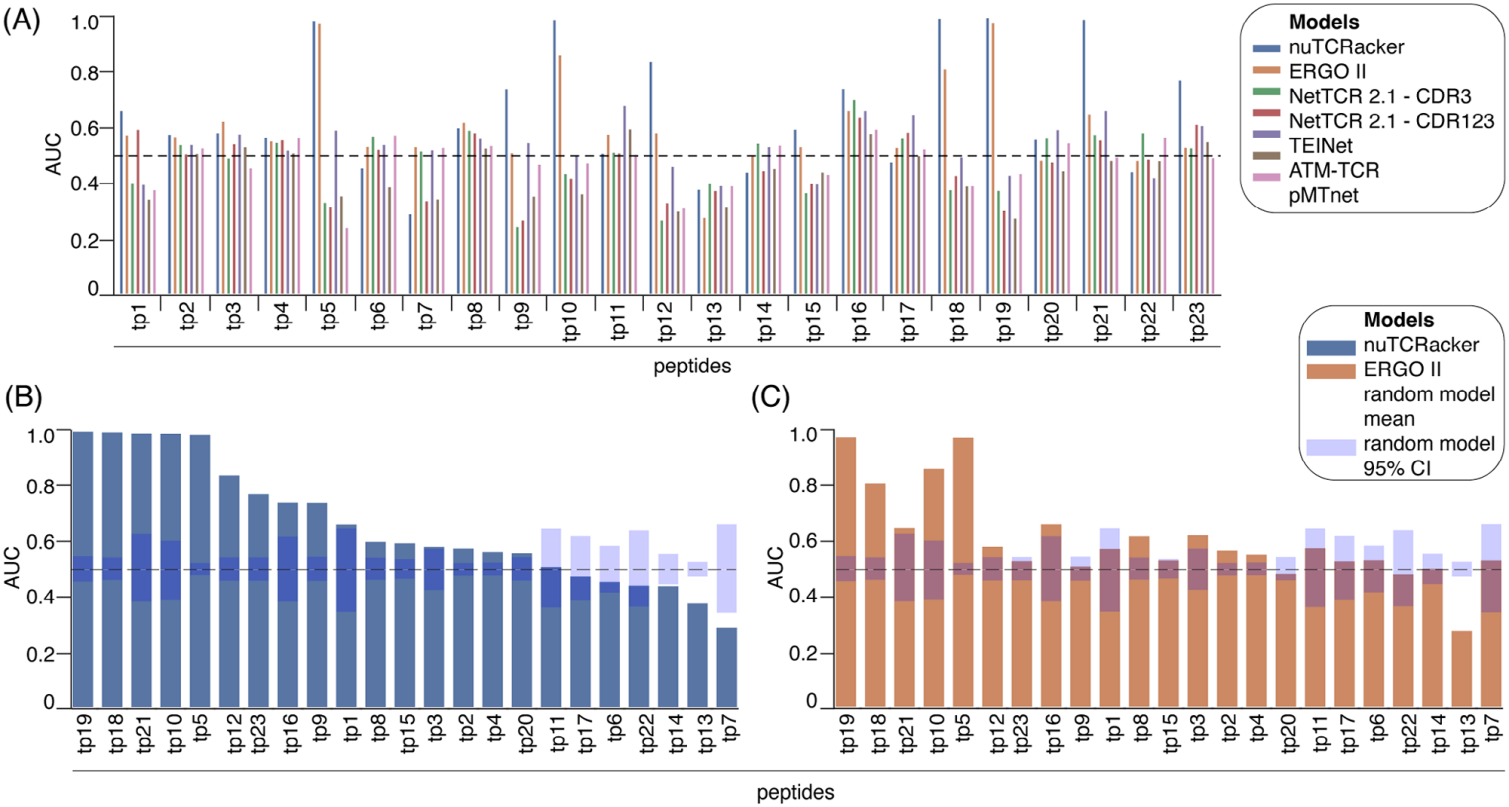
Performance of αβTCR‐peptide‐HLA‐I binding prediction algorithms. (A) The 23 individual peptides (each with at least 50 αβTCR binders/non‐binders of a given peptide) are on the *x*‐axis, with AUC on the *y*‐axis. AUC = 0.5 (representing performance no better than random) is indicated by a dashed line. (B, C) The AUC performance of nuTCRacker (B) and ERGO II (C) on the 23 peptides compared with random, ordered by decreasing AUC.

The focus of the evaluation was on assessing the ability of tools to predict whether a given αβTCR would bind to a peptide that was not present in the training set, using the labelled dataset previously prepared (Figure 1). For the purposes of this evaluation, we consider a peptide to not be present in the training set if no peptide in the training set is identical to the peptide of interest. The tool performance was evaluated using a partial leave‐one‐group‐out cross‐validation (LOGOCV) strategy whereby, at each iteration, the αβTCR‐peptide‐HLA‐I data associated with a single peptide was omitted from the training set and used exclusively for testing. However, to facilitate robust inferences about the performance associated with individual peptides, only peptides associated with at least 50 αβTCRs were tested in this way, of which there were 23 (Figure 1; Table S2). The choice of 50 cognate αβTCRs as a minimum threshold represents a trade‐off between, on the one hand, a desire to maximise the number of peptides evaluated and, on the other hand, a desire to ensure sufficient confidence in the reported performance for each individual peptide. As a measure of prediction accuracy, we used the area under the receiver operating characteristic curve (AUC) scores of all tools for each of the 23 peptides (Figure 3A). Overall, nuTCRacker was the best‐performing model, with an AUC over 0.9 for 5 peptides and over 0.7 for 9 peptides. The second‐best performing method, ERGO II, had an AUC over 0.9 for only 2 peptides and over 0.7 for only 4 peptides. For five of the peptides, no method achieved an AUC significantly better than a random model (Figure 3A).

To further investigate the performance difference among the two best‐performing methods, random model confidence intervals (CIs) were calculated as follows: (1) the prediction for each record in the LOGOCV test set for a given peptide was drawn from a random uniform distribution [0,1] and the corresponding test AUC calculated; (2) this process was repeated 1000 times using different random seeds, yielding 1000 AUC point estimates for each of the 23 peptides; and (3) these peptide‐level distributions were used to calculate the mean and 95% confidence interval values (Figure 3B,C). As expected, the mean of the random models for every peptide was close to 0.5, and the differences in the size of the confidence intervals associated with different peptides are highly correlated with the number of αβTCRs associated with each peptide (Table S2). Statistical significance was estimated by calculating *p*‐values using non‐parametric bootstrapping (*n* = 10,000) applied to the AUC performance distributions of nuTCRacker and ERGO II on the 23 peptides and compared with random model performance, indicating that nuTCRacker performed significantly better than random for 16 out of 23 peptides and ERGO II significantly better than random for 12 of the 23 peptides. For five of the peptides, no method achieved an AUC significantly better than a random model (Figure 3A). It is interesting to note that all the evaluated tools performed significantly worse than random with peptide [RAKFKQLL] (tp13; see Table S1), which was the only peptide out of the 23 with a length of 8 residues.

**FIGURE 4:**
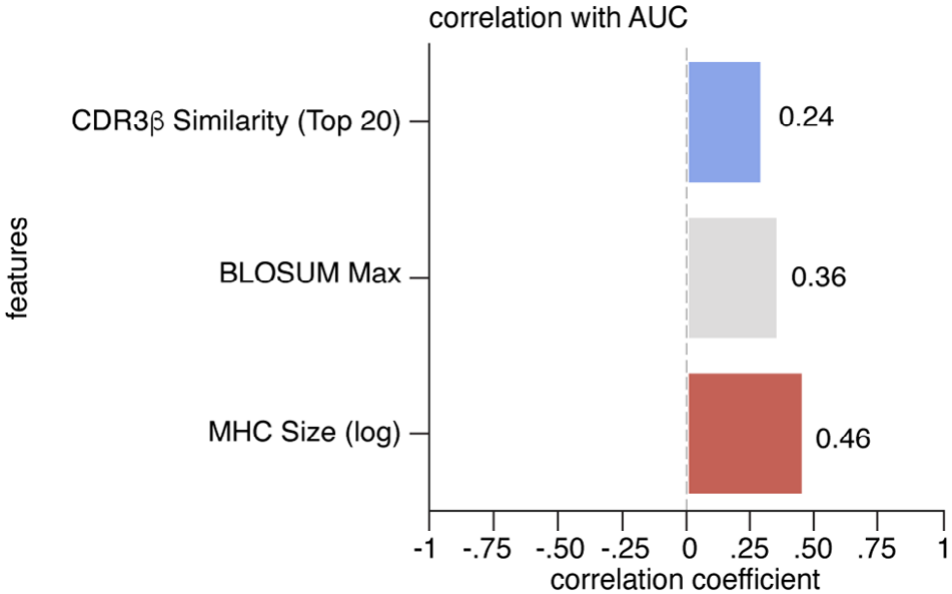
Matrix showing the correlation between AUC and the three measures of training/test relationships that showed the stronger correlation for nuTCRacker. The correlation coefficient of the three features showed the strongest correlation with the AUC of nuTCRacker. Pearson correlation test was applied. A matrix incorporating a more extensive set of properties is shown in Figure S2. Correlation coefficient values are also reported as numbers for each feature.

Given that nuTCRacker gave accurate predictions for some unseen peptides (a subset of which were also accurately predicted by ERGO II), but not others, we investigated the underlying factors that could explain why certain peptides were more tractable than others. No single property that we investigated had a Pearson correlation with AUC above 0.5 (Figure S1), although partial correlations were observed for properties associated with each of the three physical components (αβTCR, HLA‐I and peptide) forming a αβTCR‐peptide‐HLA‐I complex (Figure 4; Figure S2). The following properties stood out: (1) for αβTCRs, the degree of similarity between the target CDR3β and the most similar CDR3β sequences in the training set, with similarity measured using TCRdist3; (2) for HLA‐I alleles, the number of training set samples (taking the log of that number) having the same HLA‐I allele as the target HLA‐I complex; (3) for the peptides, the distance between the target peptide and the most similar peptide measured using the aligned BLOSUM62 score. Broadly similar, though generally weaker, correlations were observed for ERGO II (Figure S3).

To gain practical insights into the type and number of samples associated with high‐accuracy predictions for an unseen peptide, a decision tree was generated from the predictions made by nuTCRracker using scikit‐learn. For nuTCRacker, the most important determinant of success in out‐of‐distribution peptide prediction was having several hundred examples in the training set from the same HLA‐I allele (although the number of HLA‐I alleles for which we had sufficient examples was too small to derive general and precise guidance).

### 2.4 Application of nuTCRacker and Competing Models to an in Cellula‐Validated Dataset of αβTCR‐Peptides Associated with Cancer and Not Previously Seen by the Models

One possible bias in the training dataset used above could have been the promiscuity of immunology methods and statistical approaches used to define whether a given αβTCR bound a given peptide‐HLA‐I complex in the various studies that provided the input for the training dataset. To address this issue, we generated quantitative data measuring the recognition of αβTCRs and peptide‐HLA‐I complexes and the downstream activation of the cell expressing the αβTCRs upon binding to the specific peptide‐HLA‐I complex. To this end, we cloned the CDR3, V and J regions of each αβTCR into Jurkat triple reporter (Jurkat‐TPR) cells, a cell line engineered to express reporter genes NFAT‐eGFP, NF‐κB‐CFP and AP‐1‐mCherry, which are induced upon cell activation by αβTCR recognition of peptide‐HLA‐I complexes [43]. NFAT, NF‐κB and AP‐1 are transcription factors that regulate T‐cell cytotoxicity [44, 45].

**FIGURE 5:**
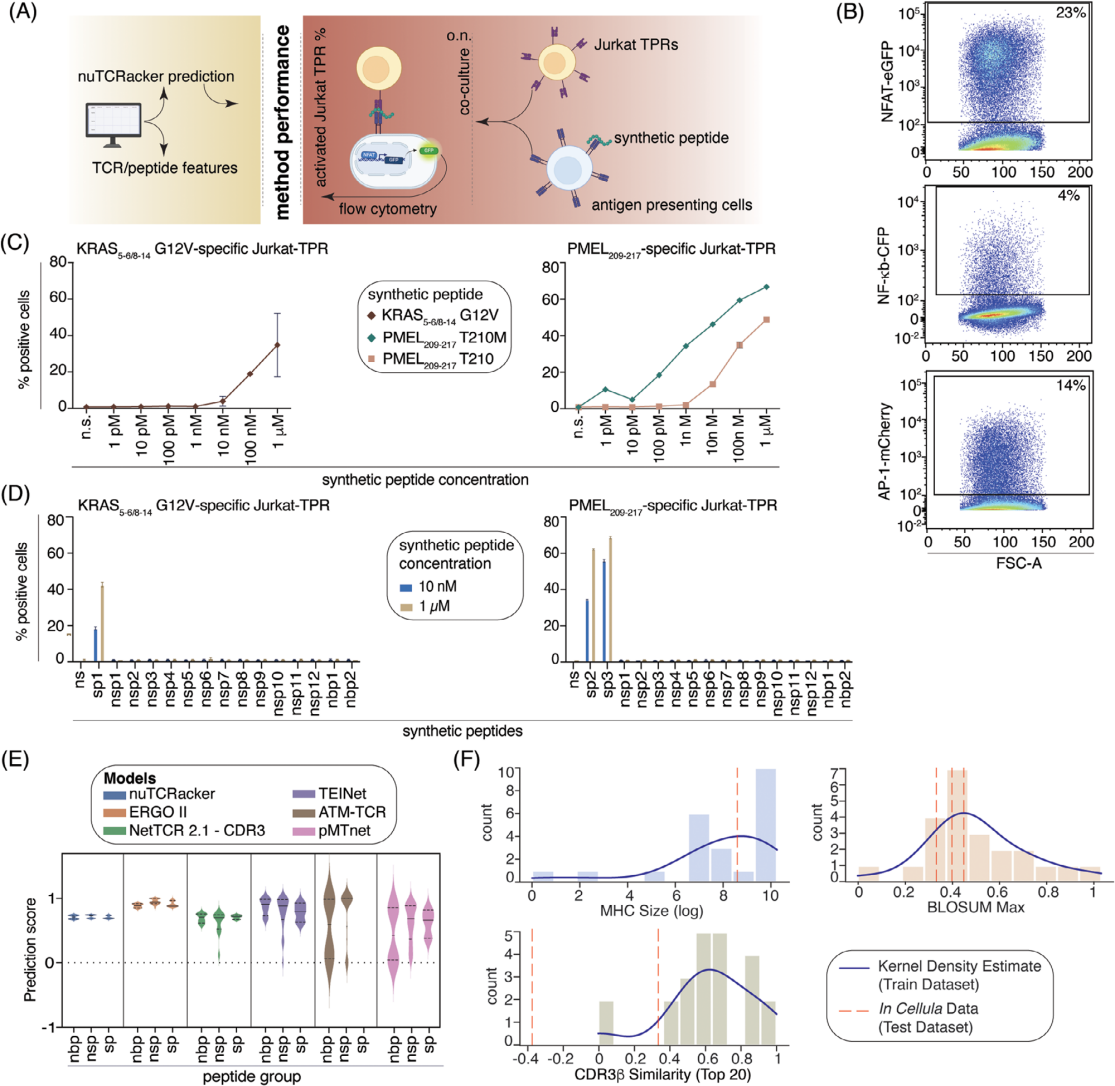
Predictors’ performance on an in cellula‐validated dataset of cancer‐associated αβTCR‐peptide‐HLA‐I. (A) Experimental design. (B) Representative measurement of the activation of the three markers of the αβTCR‐transduced Jurkat‐TPRs upon co‐culture with T2 cells pulsed with the specific synthetic peptide. Here we show the activation of KRAS_5‐6/8‐14_‐specific αβTCR‐transduced Jurkat‐TPRs upon co‐culture with T2 cells pulsed with 1 µM of the KRAS_5‐6/8‐14_ [KL][VVGAVGV] peptide, as detected by measuring the NFAT‐eGFP, NF‐κB‐CFP, and AP‐1‐mCherry activation markers. (C) Frequency of activated αβTCR‐transduced Jurkat‐TPRs upon co‐culture with T2 cells pulsed with different synthetic peptide concentrations. The NFAT‐eGFP marker was used for measuring the cell activation. Mean and SD (bars) of 2 technical replicates are reported for the synthetic peptides PMEL_209‐217_ T210 [ITDQVPFSV] and KRAS_5‐6/8‐14_ [KL][VVGAVGV]. For the PMEL_209‐217_ T210M [IMDQVPFSV], a single assay was carried out, although the result was confirmed using a different cell background (i.e., K562‐A*02:01 cell line, see Figure S4). (D) Frequency of activated αβTCR‐transduced Jurkat‐TPRs upon co‐culture with T2 cells pulsed with synthetic peptides either known to be recognised by the αβTCRs (sp) or predicted to bind the HLA‐A*02:01 (nsp) or predicted to not bind the HLA‐A*02:01 (nbp). The full peptide sequence is reported in Table S3. The NFAT‐eGFP marker was used for reporting the cell activation. The mean and SD (bars) of 2 technical replicates are reported. The KRAS_5‐6/8‐14_‐specific and PMEL_209‐217_‐specific Jurkat‐TPRs were transduced at 50% and 75% transduction efficiency, respectively. (E) Prediction of αβTCR‐peptide‐HLA‐I binding for peptides belonging to groups sp, nsp and nbp by nuTCRacker and the other 5 algorithms benchmarked. The AUC is reported as a measure of prediction accuracy. (F) Distributions of TCR, peptide, and HLA‐I similarity between the training dataset and in cellula test dataset, with vertical dashed lines representing the feature values associated with the 2 αβTCRs, 3 peptides, and one HLA‐I allele found in the in cellula dataset, respectively. The TCR and peptide distances would suggest that the model should not be expected to make accurate predictions for these αβTCR‐peptide‐HLA‐I.

We co‐cultured the αβTCR‐transduced Jurkat‐TPR with T2 cells presenting selected synthetic peptides onto the cognate HLA‐A*02:01 complex (Figure 5A). We selected 2 αβTCRs described to specifically recognise either the cancer‐associated non‐spliced epitope PMEL_209‐217_ [ITDQVPFSV] and its variant PMEL_209‐217_ T210M [IMDQVPFSV] [46, 47] or the putative cancer‐specific *cis*‐spliced epitope KRAS_5‐6/8‐14_ [KL][VVGAVGV] [48]. The non‐spliced epitope PMEL_209‐217_ is a well‐known melanoma‐associated epitope that has been used as a target in several immunotherapy trials [49, 50, 51], and its production via antigen presentation pathways has been extensively investigated [47, 52, 53, 54, 55]. The αβTCR able to recognise the putative cancer‐specific *cis*‐spliced epitope KRAS_5‐6/8‐14_ is the first example of αβTCR specific for an HLA‐A*02:01‐restricted epitope candidate carrying one of the most recurrent driver mutations of pancreatic adenocarcinoma. The physiological antigenicity and proteasome‐dependent production of this epitope candidate have been debated [48, 56, 57].

Upon co‐culture with T2 cells pulsed with the target synthetic peptides, all three activation markers of the αβTCR‐transduced Jurkat‐TPRs were triggered, as measured by flow cytometry (Figure 5B), in agreement with the original study describing this cellular system [43]. The activation of the αβTCR‐transduced Jurkat‐TPRs was dependent on the concentration of synthetic peptide‐pulsed to T2 cells (Figure 5C). As proof of principle, this dependency between the peptide amount and the Jurkat‐TPC activation was confirmed by co‐culturing the latter with another antigen‐presenting cell line, that is, the K562‐A*02:01 cell lines pulsed with the synthetic peptide PMEL_209‐217_ T210M [IMDQVPFSV] (Figure S4).

To preliminarily investigate the association between the Jurkat‐TPR activation and the peptide‐HLA‐I binding affinity in our experimental setup, we co‐cultured the Jurkat‐TPR with T2 cells pulsed with different concentrations of two very similar synthetic peptides that were recognised by the same αβTCR, that is, the synthetic peptide PMEL_209‐217_ T210 [ITDQVPFSV] and PMEL_209‐217_ T210M [IMDQVPFSV]. The activation triggered by the T2 cell pulsed with the synthetic peptide PMEL_209‐217_ T210 [ITDQVPFSV] was weaker than what was measured by pulsing T2 cells with the same concentration of the PMEL_209‐217_ T210M synthetic peptide (Figure 5C). This is in agreement with previous studies showing a stronger binding affinity of the latter peptide (IC_50_ = 19 nM) than the former (IC_50_ = 172 nM) for the HLA‐A*02:01 complex [46], as confirmed using other strategies [47, 58]. The titration curve shown in Figure 5C confirmed that this assay allowed a quantitative output, wherein we could not only discriminate between αβTCR binding and nonbinding of a given peptide‐HLA‐I complex but also how much a given αβTCR‐peptide‐HLA‐I complex could activate αβTCR‐expressing cells. Indeed, by pulsing T2 cells with synthetic peptides with different concentrations and measuring the activation of the αβTCR‐transduced Jurkat‐TPRs, we observed a direct quantitative correlation between the amount of target epitope bound to the HLA‐I complexes at the cell surface and the Jurkat‐TPR's activation (Figure 5C), which is mediated by the αβTCR‐peptide‐HLA‐I interaction. Furthermore, two peptides bound to the same HLA‐I and recognised by the same αβTCR could be different in Jurkat‐TPC activation in a quantitative fashion, depending on the peptide‐HLA‐I binding affinity. However, it is worth noting that this kind of experiment could not discriminate between αβTCR‐peptide‐HLA‐I binding affinity and peptide‐HLA‐I binding affinity.

We then tested these two αβTCRs‐transduced Jurkat‐TPRs by co‐culture with T2 cells pulsed with three synthetic peptide groups containing: the recognised peptide(s) (sp), 14 peptides predicted to bind the HLA‐A*02:01 complex (nsp) and 2 peptides not predicted to bind the HLA‐A*02:01 complex (nbp) (Table S3). We used two synthetic peptide concentrations (i.e., 10 nM and 1 µM) to generate a quantitative dataset for all tested peptide groups. The two αβTCRs recognised the known target synthetic peptides quantitatively and none of the peptides that were assumed to not be recognised (Figure 5D). We then applied nuTCRacker and the other five models to a dataset of peptide sequences containing three synthetic peptide groups and the two αβTCRs. None of the models were able to discriminate between the three peptide groups (Figure 5E).

To understand which features of the αβTCR‐peptide‐HLA‐I of this dataset may have made prediction difficult, we compared the features of the αβTCR‐peptide‐HLA‐I contained in this dataset with those present in the training dataset. Based on the correlations noted above and in Figure S1, we posit that the sequence similarity between the αβTCRs of this dataset and those present in the training dataset as well as the sequence distance between the peptides in this dataset and those in the training set stand out as likely contributing factors (Figure 5F).

## 3 Discussion

In this research we have addressed the generalised αβTCR‐peptide‐HLA‐I binding prediction task, that is, the prediction of αβTCR‐peptide‐HLA‐I binding of peptides that was not included in the training dataset, which is one of the most challenging objectives of the current research in this specific field. To this end, we developed our own, novel deep learning method nuTCRacker, and we have benchmarked its performance, together with the performance of five existing state‐of‐the‐art methods, using a large, curated dataset that we created for this purpose. The nuTCRacker incorporated various features that are new to the prediction of αβTCR‐peptide‐HLA‐I binding, such as transfer learning at scale (widely used in natural language processing). Our results suggest that the prediction of αβTCR binding for unseen peptides is more tractable than has been assumed, with a cross‐validation AUC greater than 0.7 achieved for 9 out of 23 unseen peptides. To make a fair comparison with existing methods, five were retrained using our datasets and none performed as well as nuTCRacker, with the second‐best predictor, ERGO, achieving a cross‐validation AUC greater than 0.7 for only 4 of the 23 unseen peptides. The predictions obtained by nuTCRacker show that although some improvement was achieved with our strategy, the generalised αβTCR‐peptide‐HLA‐I binding prediction remains an untangled knot for most of the peptides, as it is for the other predictors benchmarked in this study. Once this knot is untangled further levels of complexity such as the impact that PTMs can have on αβTCR‐peptide‐HLA‐I binding and CD8^+^ T cell activation could be addressed.

Whereas previous work has estimated that 50–200 cognate αβTCRs were required for a peptide of interest [20, 21], our analysis suggested that successful predictions for unseen peptides depend on three primary factors: (1) representation of the associated HLA‐I allele (preferably several hundred samples); (2) inclusion of at least one peptide with sequence similarity; and (3) training data containing αβTCRs with sequence similarity to the target TCR.

The interplay between these factors requires further exploration, which would necessitate more data.

### 3.1 Data Limitations and Perspectives

There are several limitations in the publicly available data for this task which hamper the ability of models to learn the true underlying biology of αβTCR‐peptide‐HLA‐I recognition. First, the lack of experimental negative data necessitates the simulation of negative data via mispairing of TCRs with peptide‐HLA‐I. This presumes no cross‐reactivity, but perhaps more problematic is that it creates biases in the negative set which may not be present in experimentally determined non‐binders. Second, due to the unbalanced nature of the dataset with respect to peptides, we chose to down‐sample the αβTCRs associated with a single CMV epitope dominating the dataset. This was a trade‐off leading to a suboptimal underutilisation of data that might be underestimating the predictive value of a highly immunogenic epitope. Finally, the literature‐based dataset used for the development and benchmark of nuTCRacker had a binary (qualitative) input referring to the binding of αβTCRs and peptide‐HLA‐I, that is, specific αβTCRs and peptide‐HLA‐I were defined as either binders or non‐binders in the original experiments.

As the future improvement of nuTCRacker and other methods, we estimate that quantitative datasets could be used, that is, those derived from in cellula experiments that not only define if a given αβTCRs can recognise a peptide‐HLA‐I complex but also what is the avidity of the αβTCRs for the peptide‐HLA‐I complex. The dataset used in Figure 5 is this kind of dataset, which was generated by using a Jurkat‐TPR cell line and quantitatively measuring their activation by flow cytometry. Of course, this specific dataset, which was generated as proof of principle in this study, was too small to be a proper validation dataset and was restricted to a specific HLA‐I allele, that is, the HLA‐A*02:01. A significantly larger number of peptides and αβTCRs, as well as HLA‐I alleles would be needed for future developments of generalized αβTCR‐peptide‐HLA‐I binding prediction. Other experiment read‐outs that could be used to this end include but are not limited to, IFN‐γ ELISAs, cytotoxicity assays, or flow cytometry to ascertain levels of activation and degranulation markers, such as CD107 [17, 59, 60].

It is also worth noting that the vast majority of publicly available data characterise the binding of αβTCRs to peptides derived from viral antigens. There are few examples of binding to peptides derived from cancer neoantigens. It is possible that the dynamics of these subclasses differ and that models trained on the viral subclass would not generalise to the cancer‐associated antigen subclass. Unfortunately, there is not enough data for the latter presently to support a robust analysis of this kind of cross‐subclass transfer learning.

## 4 Materials and Methods

### 4.1 Dataset Preparation for Method Cross‐Validation

#### 4.1.1 Labelled Data

αβTCR‐peptide‐HLA‐I binding data were downloaded from five public databases and repositories: VDJdb [27], McPAS‐TCR [28], TBAdb [29], IEDB [30] and 10x Genomics. Downloaded data were then filtered to exclude records that failed to meet the following requirements for a suitable αβTCR‐peptide‐HLA‐I complex: the TCR has an α and/or β chain comprising a specified CDR3 sequence and assigned V and J genes; the peptide has a length of 8 to 12 residues; the HLA‐I molecule is of human origin and the HLA type is specified to at least the allele group level; and there are no non‐standard or ambiguous amino acids in the peptide or CDR3. Care was taken to exclude records mislabelled as human (e.g., those with mouse MHC allele H‐2Kb) or as MHC class I (e.g., those with MHC labels containing codes such as ‘DRB’ that are associated with MHC‐II nomenclature). The small number of HLA‐I allele labels that were specified only to the allele group level (e.g., HLA‐A*02) were manually assigned to a specific HLA‐I allele based on peptide‐HLA‐I binding data from other sources, notably the training set provided with NetMHCpan 4.1 [26]. Where no such evidence was found, allelic assignments were made according to global HLA‐I allele frequencies based on data from the Allele Frequency Net Database [61] (e.g., in the case of the allele group HLA‐A*02, the most frequent allele is HLA‐A*02:01). α and β chain V gene annotations that contained only the gene name were manually assigned the appropriate allele name by imputation, for example by inferences based on a comparison of the given sequence information (notably the start of CDR3) with the canonical TCR reference sequences provided by IMGT [62].

More complicated strategies were required for handling IEDB and 10x Genomics data. With respect to IEDB, having made an initial selection of T cell assay data associated with human hosts and linear epitopes with MHC class I restriction, both “calculated” data (in which CDR3 locations are inferred via automated analysis of TCR sequences) and “curated” were coalesced to maximise the number of complete records, with missing values in the “calculated” data (*e.g*., V and J alleles of TCR α and β chains) imputed from the “curated” data where possible. Data from VDJdb, McPAS‐TCR and TBAdb were used to impute missing HLA‐I alleles for peptides in the IEDB data.

With respect to 10x Genomics, 4 BEAM‐T datasets and 4 multiplex datasets were downloaded. Cells were filtered to include only productive, high‐confidence, full‐length αβTCR T cells with completely inferred V and J genes. Cells were further filtered to include only those with exactly two distinct TCR chain sequences in order to remove dual‐receptor T cells and experimental artefacts (notably doublets and GEMs without cells). Lastly, gene expression data was converted to binary binding flags (*i.e*., binding/non‐binding) by defining a binding threshold as follows: a given αβTCR‐peptide‐HLA‐I complex is considered a binder if the associated peptide UMI count is greater than three standard deviations higher than the UMI count for the experiment's negative control peptide, where the standard deviation is calculated based on the UMI counts for the negative control peptide across all cells in the experiment. This approach was adopted prior to the publication of ITRAP [63]—noted for its “overall superior performance” in a recent benchmark study [64]—but in practice, our protocol was found to produce near‐identical filtering choices. Only positive binders were selected for our dataset; negative (nonbinding) samples are discussed further.

Across all the preceding datasets, relevant fields were converted to a single, standardised format, all records were combined in a single set, and duplicates were removed. Negative data were created by simulating nonbinding αβTCR‐peptide‐HLA‐I complex pairs via the random mispairing of αβTCR and peptide‐HLA‐I complexes from this aggregated dataset, a strategy widely adopted in other studies; given that a small number of αβTCRs appear in multiple samples bound to different peptide‐HLA‐I complexes, the simulated negatives were screened to exclude any true positives generated at random. This approach was preferable to the alternative strategy of randomly pairing epitopes with αβTCRs derived from a disjoint source (e.g., one using different experimental techniques), as the latter conferred a bias to the negative data that could be utilised by advanced machine learning methods to misleadingly boost performance.

It is also worth noting that, although the 10x Genomics dataset contained many experimentally confirmed negatives, these are from only a small number of individuals, so of limited diversity.

Given that more than 50% of the positive samples were associated with a single CMV epitope, these were down‐sampled to give approximately the same number as that associated with the second most frequent epitope, giving a total of 2200 positive samples. An equal number of negative samples was generated, giving a total of 32,440 αβTCR‐peptide‐HLA‐I complex samples in our dataset. Note that downsampling the CMV epitope was not motivated by balancing the epitope classes but rather to enable the random sampling of these simulated negative examples without replacement as the CMV set made up more than 50% of the dataset. Downsampling the CMV set enabled us to have an equivalently sized negative pool to sample CMV negatives. This 50/50 ratio of negatives to positives was preferred to a higher proportion of negatives because our chosen methodology is insensitive to this ratio, training times are shorter, and generating a large number of additional negative samples would entail additional downsampling of positive samples associated with epitopes that are highly represented. However, it is certainly true that the number of non‐binding αβTCR‐peptide‐HLA‐I complex combinations within T cell repertoires greatly exceeded the number of binding combinations, and that additional negatives had the potential to provide additional useful information. In practice, several tools (e.g., ERGO‐II [38], NetTCR‐2.1 [21, 39], pMTnet [40]) have been developed with a higher, though essentially arbitrary, proportions of negative samples. We undertook some additional evaluations to test whether additional negatives improved predictive performance.

It is worth acknowledging that the final dataset retained a significant level of bias with respect to the prevalence of different HLA‐I alleles, with HLA‐A*02:01 and HLA‐A*03:01 accounting for 36.4% and 26.2% of the samples, respectively.

#### 4.1.2 Unlabelled Data

To explore the potential benefits of adopting transfer learning approaches in the context of αβTCR‐peptide‐HLA‐I complex binding prediction, large corpora of unlabelled data*—*both αβTCRs of unknown peptide specificity and peptide‐HLA‐I data with unknown cognate αβTCRs—were collated.

Bulk TCR repertoire data were downloaded from iReceptor [3]. Sequences were filtered for organisms matching “Homo sapiens” (NCBITAXON:9606) and PCR targets “TRA” or “TRB”. All sequences matching these criteria were downloaded, encompassing repertoires from the VDJServer [65] and AIRR COVID‐19 [66] repositories as well as the iReceptor Public Archive. The resulting combined dataset was filtered to include only productive sequences from locus TRA or TRB with specified CDR3, and V and J genes. Paired sequences were inferred where there were exactly two sequences (one from the TRA locus, one from the TRB locus) with identical cell ID and repertoire ID. Duplicate sequences were removed, resulting in 410,499 paired and 24,429,131 unpaired TCR sequences.

Additional bulk TCR data were downloaded from 10x Genomics (10x Genomics, 2023). Data from 21 studies were downloaded and combined into a single dataset. This dataset includes T cells derived from peripheral blood and bone marrow of donors either with melanoma, CMV infection, non‐small‐cell lung cancer (NSCLC) or healthy donors. The resulting dataset was filtered to include only records which were flagged as productive cells of high confidence. Within each sample, records were filtered so that a single barcode was associated with exactly two records to ensure that multiplets, double α cells and other comparable edge cases were removed. For each barcode the associated TRA and TRB records were merged, resulting in 201,094 paired sequence records.

Finally, with respect to the peptide‐MHC component of the αβTCR‐peptide‐HLA‐I complex, bulk data were downloaded from the training set provided with NetMHCpan 4.1 [26] and filtered to include only peptide‐HLA complexes associated with human HLA‐I alleles and where αβTCR‐peptide‐HLA‐I complex binding has been experimentally confirmed.

#### 4.1.3 Sequence Preparation

Typically, our approaches require access to either full TCR amino acid sequences or to all their CDR subsequences, whereas many TCR binding data repositories store only a minimal description of a given TCR consisting of its CDR3 sequences and its V and J gene identifiers. In such cases, Stitchr [31] was used to reconstitute the complete α and β chain amino acid sequences from their minimal descriptions. For approaches requiring only CDR sequences, it is necessary (where this information is not provided by the source repository) to specify CDR start and end residues within a full α or β sequence. Here ANARCI [67] was used to number the α and β amino acid sequences and the CDR sub‐sequences identified using the positional definitions developed by IMGT [62].

In the case of HLA molecules, we explored the use of pseudosequences as a concise alternative to lengthy HLA amino acid sequences. Here an HLA pseudo‐sequence comprises the set of 34 amino acid residues specified by Nielsen and co‐workers [32] based on their analysis of residues lying within 4.0 Å of bound 9 amino acid long peptides for a representative set of HLA‐A and HLA‐B structural complexes.

### 4.2 Measuring Model Performance

For the cross‐validation strategy, we used a partial LOGOCV strategy, which involved splitting the dataset into a training set and testing set by creating a test set from one observation and using all but one observation as the training set. This process was repeated n times, where n is the number of records in the dataset. In our case, we employed a modified version of LOGOCV focused on holding out all data associated with a particular peptide to evaluate models’ abilities to make predictions on out‐of‐distribution peptides. In this modified approach, we created a test set using all records associated with a particular peptide, and a mutually exclusive training set from records associated with all other peptides. Since not all peptides had sufficient data to populate a statistically significant test set, we only created test splits for the 23 peptides in the dataset which have greater than 50 records associated with them.

Since the problem of predicting αβTCR‐peptide‐HLA‐I binding was here modelled as a binary prediction task (i.e., a given combination of αβTCR, peptide and HLA‐I allele was predicted to bind or not bind), the primary metric used for evaluation was the AUC.

### 4.3 Selection of the Published Predictor Models

A key aim of this research was to benchmark the performance of both our own and existing tools on the generalised αβTCR‐peptide‐HLA‐I binding prediction task. For an existing tool to be suitable for benchmarking, it was necessary that we could retrain it using our benchmark dataset. For a given tool, we checked that we were able to correctly replicate the authors’ training process by reproducing its published performance using the training data provided by the tool. Given that certain tools were under ongoing refinement, it was considered sufficient to achieve a close, but not necessarily identical, performance score. The retraining was considered both impractical and unnecessary for pretrained tool components that encode large datasets of generic TCR and/or epitope data.

The following tools were successfully retrained: ERGO II, which uses a combination of LSTM‐based encoding and autoencoders, and claims “high accuracy prediction” for αβTCR binding to unseen peptides [38]; two versions of 1D CNN‐based NetTCR‐2.1 one version using paired α and β CDR3 data, the other only CDR3β data [39]; pMTnet, which uses autoencoders to generate pretrained encodings of TCR CDR3β and pMHC data [40]; ATM‐TCR, which “uses a multi‐head self‐attention mechanism to capture biological contextual information and improve generalization performance” [41]; and TEINet, which uses autoencoders to generate pretrained encodings of TCR and epitope data [42] (Table S1).

### 4.4 Alternative Strategies Preliminarily Investigated to Develop nuTCRacker

Previous work demonstrated that improved predictive performance can be achieved by including more TCR sequence regions as input to the model [68]. We therefore chose to include all of the available TCR sequence regions, including V, J and CDR3 of both the α and β chains, as input to our model. In a similar fashion, we reasoned that including HLA information (as opposed to just the peptide amino acid sequence) may improve predictive performance. With these decisions in mind, there were still many available options for how to encode these sequence regions for input to the model. For example, V and J genes could be treated as categorical inputs or translated to their associated CDR sequences. Likewise, the HLA‐I allele could be treated as a categorical or sequence input.

Since we were primarily interested in out‐of‐distribution generalization, sequence‐based inputs were the obvious choice. Representations based on categorical inputs could not apply previous learning to new categorical values, whereas sequence inputs could, owing to their compositional nature.

Having decided on sequence inputs there were still choices to make regarding whether to use complete sequences or subsequences as inputs. Complete sequences (such as the full α and β chains of the TCR and the full amino acid sequence of the HLA‐I) provide the maximum input information, but given the limited amount of binding data for training, it may be more practical to provide sequence fragments (such as just the TCR CDR sequences), thereby reducing the number of parameters that must be learned by the model. We experimented with a variety of combinations of input sequences and subsequences and ultimately chose to include full α and β chain amino acid sequences, full peptide amino acid sequence and the HLA‐I pseudo sequence as the inputs to the model [32].

### 4.5 Selection of Cancer‐Related αβTCR‐Peptide Dataset for in Cellula Validation of αβTCR‐Peptide Binding

To generate an in cellula‐validated dataset of αβTCR‐peptides associated with cancer and not previously seen by the algorithms, we selected αβTCRs recognising either the cancer‐associated non‐spliced epitope PMEL_209‐217_ [ITDQVPFSV] and its variant PMEL_209‐217_ T210M [IMDQVPFSV] [46, 47] or the *cis*‐spliced peptide KRAS_5‐6/8‐14_ [KL][VVGAVGV] that carries the KRAS G12V mutation [48]. All three peptides could be presented by the HLA‐A*02:01 complex (Table S3). For the assay, these two αβTCRs were tested against three classes of peptides: the recognised peptide(s) (sp), 14 peptides predicted to bind the HLA‐A*02:01 complex (nsp) and two peptides not predicted to bind the HLA‐A*02:01 complex (nbp). The selected peptides included in the groups nsp and nbp were either viral or cancer‐associated epitopes, as outlined in Table S3. Viral epitopes comprised only those with immunogenicity validated in in vitro assays, as reported in the IEDB database [30] whereas the cancer‐associated epitope was a *cis*‐spliced epitope recognised by T cells of melanoma patients [54]. Peptide‐HLA‐I binding affinity was predicted by using NetMHCpan 4.0 BA. A predicted binding affinity (IC_50_) of 500 nM was used as a cut‐off between the group nsp and nbp. Each αβTCR‐peptide pair was tested in cellula, as detailed below. Concurrently, predictions were generated for this dataset using nuTCRacker and the five tools being benchmarked. To describe the features of this dataset in comparison to the training dataset, scores for peptide similarity and CDR3 sequence similarity were additionally calculated using BLOSUM62 substitution scores and TCRdist, respectively.

### 4.6 Synthetic Peptides

Peptides for synthesis were initially screened to predict the success of Fmoc‐based peptide synthesis [69], and then synthesized by standard Fmoc‐based peptide synthesis protocol (Pepmic).

Synthetic peptides were initially suspended at 10 mM concentration in a 10% DMSO solution and then diluted in IMDM media to concentrations ranging from 1 pM to 1 µM, as described in the specific experiments.

### 4.7 Cell Lines

Jurkat 76 TPR cells, T2 cells and K562 cells were cultured in IMDM (Gibco) supplemented with 10% FCS and 1% penicillin/streptomycin (HyClone), and incubated at 37°C with 5% CO_2_, whilst for HEK‐293T cells DMEM (Gibco) media was used.

K562‐A02 cells were generated via transduction of K562 cells with a plasmid encoding β2m‐fused HLA‐A*02:01. The Jurkat‐TPR cell system was used to express αβTCRs.

### 4.8 Plasmid Cloning and Lentiviral Transduction

Epitope‐specific TCR CDR3 α and β chain sequences were obtained from published studies, and sequences of their respective V and J regions were obtained via IMGT. A T2A cleavage site was incorporated between the alpha and beta sequences. For the HLA plasmid, the construct comprised a signal peptide, a β2m light chain sequence, 4× GGGGS linkers, and the HLA‐A*02:01 heavy chain. Codon‐optimised sequences were synthesised in a pUC57 plasmid (Genscript, UK), and subsequently cloned into a transfer plasmid belonging to a 2nd generation lentiviral system.

For transduction, HEK‐293T cells were seeded at 5 × 10^5^ cells/well. The next day, they were transfected with plasmid DNA at a 3:1 PEI:DNA ratio. After 48 h, lentiviral supernatant was harvested for a 1 h spinfection with K562 cells or Jurkat TPR cells at 1000*g*. Transduced cells were seeded in a 12‐well plate and transferred to a six‐well plate the following day. Expression of TCR was confirmed via flow cytometry staining for anti‐CD3 (BD Horizon) and anti‐TCR (BioLegend), and successful transduction of HLA‐A*02:01 into K562 was confirmed via staining for anti‐w6/32 pan‐HLA antibody (BioLegend).

### 4.9 In Cellula Assay to Measure the Synthetic Peptide Recognition by Jurkat TPR

The Jurkat‐TPR is a CD8^+^ TCR α^−^/β^−^ reporter system that expresses GFP, CFP and mCherry upon activation of the NFAT, NF‐κB and AP‐1 pathways, respectively [43]. To quantitatively evaluate αβTCR specificity/avidity to a given peptide, T2 and K562‐A*02:01 cell lines were pulsed with synthetic peptides for 1 h, and then co‐cultured with αβTCR‐transduced Jurkat‐TPRs for 16 h. An E:T ratio of 1:1 was used. The αβTCR transduction efficiency of the Jurkat‐TPR was in the range of 75% for PMEL_209‐217_‐specific Jurkat‐TPRs and 50% for KRAS_5‐6/8‐14_‐specific Jurkat‐TPRs. αβTCR‐transduced Jurkat‐TPRs were not sorted before the co‐culture assay.

The αβTCR‐transduced Jurkat‐TPRs activation was measured via flow cytometry (ZE5) and the marker NFAT‐eGFP was used as representative of αβTCR‐transduced Jurkat‐TPR activation. Assays were performed in technical replicates (*n* = 2) unless stated otherwise.

### 4.10 Statistical Analysis

All statistical tests were done in either R, Python (SciPy library) or Prism v9. The Pearson correlation coefficient was used for the correlation analysis. Kernel density estimates use the Scott bandwidth method. Best fit lines use statsmodels for linear regression and show a shaded 95% confidence interval.

### 4.11 Software

Flow cytometry data were analysed using FlowJo (Treestar v10.10.0). Figures postprocessing was done with Adobe Illustrator v28.4.1. For in‐house software, see the Code Availability section.

## Author Contributions

All authors developed the project and wrote the manuscript. Justin Barton developed nuTCRacker, Trupti Gore reproduced and benchmarked the selected models, and both Justin Barton and Trupti Gore prepared the training dataset under the supervision of Adrian Shepherd. Meghna Phanichkrivalkosil performed all wet lab assays and data analysis on the TCRs and peptides used in the model application under the supervision of Michele Mishto.

## Conflicts of Interest

The authors declare no conflicts of interest.

## Peer Review

The peer review history for this article is available at https://publons.com/publon/10.1002/eji.202451607.

## Supporting information




**Supporting Information file 1**: eji5995‐sup‐0001‐SuppMat.pdf

## Data Availability

TCR‐peptide‐HLA binding data were downloaded and collated from VDJdb [27], McPAS‐TCR [28], TBAdb [29], IEDB [30] and 10x Genomics. The αβTCR‐peptide‐HLA‐I dataset created for training, testing and cross‐validation is available upon request and depending on the approval of the authors who generated the original datasets. Code availability: All the models and datasets are available on our lab huggingface hub here: https://huggingface.co/shepherdgroup. For example, *Fullseq model*: https://huggingface.co/shepherdgroup/fullseq‐16L8H/tree/main. *Fullseq dataset*: https://huggingface.co/datasets/shepherdgroup/fullseq/tree/main
