# Supplementary material for: nuTCRacker: Predicting the Recognition of HLA‐I–Peptide Complexes by αβTCRs for Unseen Peptides
Source: Eur J Immunol. 2025 Jul 9;55(7):e51607. doi: 10.1002/eji.202451607 (PMC12238841; doi:10.1002/eji.202451607)
Supplement: Supplementary file 1 — Supporting Information file 1: eji5995‐sup‐0001‐SuppMat.pdf [file EJI-55-e51607-s001.pdf]

nuTCRacker: predicting the recognition of HLA-I–peptide complexes by αβTCRs for unseen peptides

$ Correspondence to:
Michele Mishto (michele.mishto@kcl.ac.uk) & Adrian Shepherd (adrian.shepherd@bbk.ac.uk).

Table S1	Published αβTCR-peptide-HLA-I Prediction Tools (not an exhaustive list).
Table S2	List of peptide-HLA-I included in the test dataset
Table S3	Synthetic peptides tested with the αβTCRs-transduced Jurkat-TPR system
Figure S1	Three properties most correlated with model prediction accuracy
Figure S2	Extended matrix showing the correlation between AUC achieved by the nuTCRacker method and various features relating the training and test sets
Figure S3	Extended matrix showing the correlation between AUC achieved by the ERGO II method and features relating the training and test sets
Figure S4	Synthetic peptide concentration dependency of the $PMEL_{209\text{-}217}$-specific αβTCR-transduced Jurkat-TPR activation upon co-culture with K562-A*02:01

Name	Model architecture	Antigenic peptide encoding	TCR Encoding	TCR data	TCR V/J encoding	MHC-I encoding
ERGO II [1]	Autoencoder, LSTM	LSTM based Embedding	Autoencoder and LSTM-based embedding.	CDR3β, optional α CDR3s	Categorical embedding (fixed dimensional learned embedding vector)	Categorical embedding (fixed dimensional learned embedding vector)
pMTnet [2]	Transfer Learning, CNN	Blosum 50 + LSTM-based encoding (Encoding to a fixed-size representation)	β, Atchley factors + autoencoder based encoding	CDR 3β	n/a	34 aa pseudo-sequence+ Blosum 50 + LSTM-based encoding (Encoding to a fixed size representation)
NetTCR 2.1 [3]	1D CNN	9-mers only, BLOSUM50 encoding	BLOSUM50 encoding (zero-padding to 30)	β and/or α CDR1,2,3	Gene information in the form of CDR 1 and 2	HLA-A*02:01 only (not used)
TEINet [4]	FCN	Pretraining: epitope sequences collected from [5] using an autoencoder	Pretraining: CDR3β collected from Emerson data using an autoencoder.	CDR3β	n/a	n/a
ATM-TCR [6]	Multi-head Self Attention model	One hot vector + Contextual embedding with multi-head attention	One hot vector + Contextual embedding with multi-head attention	CDR3β	n/a	n/a
epiTCR [7]	Random Forest with 5-fold cross-validation	Blosum 62	Blosum 62	CDR3β + peptide	none	34 aa pseudo-sequence + Blosum 62
TCR-BERT [8]	Modified BERT, SVM		Pre-training: v1: β + α TCR Sequences (human & mouse). V2 using ESM-1b transformer [9]	CDR3β	n/a	n/a
TCRBert (Yoo et al., 2024)[10]	BERT	Concatenating CDR3 β+Peptide and encoded into tokens with a tokenizer, generating embeddings with a pre-trained TAPE model.		CDR3β	n/a	n/a
TITAN (Weber et al., 2021)[11]	CNN,Bimodal context Attention	Blosum 62 + SMILES	Blosum 62	CDR3β	n/a	n/a
DeepTCR [12]	VAE	n/a (in downstream analysis, the antigen specific TCRs are clustered together)	VAE-based Learned embedding	CDR3α or β	Categorial one hot representation + learned embedding	n/a
TCellMatch [13]	RNN	One hot encoding, Blosum50 and learned embedding	One hot encoding, Blosum50 and learned embedding	CDR3α and β	n/a	n/a
ImRex [14]	CNN	Image or pixel map of size 20x11x4 of CDR3β and epitope pair is encoded by physicochemical properties.		CDR3β	n/a	n/a
TCRAI [15]	CNN	In the binomial mode, the binders are the TCRs binding to a particular HLA-I-peptide.	One hot encoding + learned embedding	CDRα + CDRβ	Categorical numerical representation + learned embedding	n/a
TCRex [16]	Random Forest	Atchely Factors	Atchley Factors	CDR3 β	One hot encoding	n/a
TCRGP [17]	Gaussian Process	Peptide specific models	Blosum 62	CDR 1,2,2.5 and 3 α + β	Gene information in the form of CDR 1 and 2	n/a
ATMTCR [18]	Attention-aware contrastive learning	Blosum 50 + LSTM based embedding	Word embedding + positional embedding with sine and cosine encoding	CDR3β (datasets curated by [2])	n/a	MHC pseudo sequences + Blosum 50 + LSTM-based embedding
tcrformer [19]	Multiclass Classification with Transfer Learning-Pretrained Transformer models + finetuning	Tokenizer-based Embedding of Combined input of peptide+TCR consisting of (Input IDs, Attention Mask and special tokens)		CDR3β	Ordinal Encoder	n/a

Table S1. **Published αβTCR-peptide-HLA-I Prediction Tools (not an exhaustive list).**

Peptide name	Peptide sequence	Matched HLA-I allele	Number of TCRs associated with each peptide	Original dataset
Tp1	VLFGLGFAI	HLA-A*02:01	30	McPAS/IEDB
Tp2	KLGGALQAK	HLA-A*03:01	4,200	VDJdb
Tp3	ATDALMTGF	HLA-A*01:01	129	VDJdb
Tp4	AVFDRKSDAK	HLA-A*11:01	1851	VDJdb
Tp5	GILGFVFTL	HLA-A*02:01/HLA-A*02:01:48	2091	VDJdb/IEDB/McPAS/10x Beam-T/10x Multiplex
Tp6	DATYQRTRALVR	HLA-A*68:01	100	VDJdb
Tp7	FEDLRVLSF	HLA-B*37:01:10	28	VDJdb/IEDB
Tp8	RLRAEAQVK	HLA-A*03:01	436	VDJdb/10x Multiplex
Tp9	LLWNGPMAV	HLA-A*02:01	418	VDJdb/IEDB
Tp10	RFPLTFGWCF	HLA-A*24:02	61	VDJdb/McPAS/IEDB
Tp11	CTELKLSDY	HLA-A*01:01	33	VDJdb/IEDB
Tp12	YLQPRTFLL	HLA-A*02:01:48/HLA-A*02:01	450	VDJdb
Tp13	RAKFKQLL	HLA-B*08:01	1,231	VDJdb
Tp14	CINGVCWTV	HLA-A*02:01	245	VDJdb
Tp15	IVTDFSVIK	HLA-A*11:01	727	VDJdb/IEDB/10x Multiplex
Tp16	GPRLGVRAT	HLA-B*07:02	51	VDJdb
Tp17	RLPGVLPRA	HLA-A*02:01	48	VDJdb/IEDB
Tp18	NLVPMVATV	HLA-A*02:01/HLA-A*02:01:98/HLA-A*02:01:110/HLA-A*02:01:59	484	VDJdb/IEDB/McPAS/10x Beam-T/10x Multiplex
Tp19	GLCTLVAML	HLA-A*02:01/HLA-A*02:01:48	385	VDJdb/IEDB/McPAS/10x Beam-T/10x Multiplex
Tp20	SPRWYFYYL	HLA-B*07:02	442	VDJdb/IEDB
Tp21	RPPIFIRRL	HLA-B*07:02/HLA-B*07:02:48	49	VDJdb/IEDB
Tp22	SLFNTVATLY	HLA-A*02:01	38	VDJdb
Tp23	ELAGIGILTV	HLA-A*02:01/HLA-A*02:01:48	469	VDJdb/IEDB/10x Multiplex

Table S2. List of peptide-HLA-I included in the test dataset

Synthetic peptide name	Synthetic peptide sequence	Predicted IC_{50} for HLA-A*02:01 complex (nM)
sp1	KLVVGAVGV	34.2
sp2	ITDQVPFSV	119.5
sp3	IMDQVPFSV	4.4
nsp1	KLVVVGAVGV	174.9
nsp2	FLLNKEMYL	2.7
nsp3	YLQPRTFLL	4.3
nsp4	FIAGLIAIV	6.6
nsp5	GILGFVFTL	7.3
nsp6	LLLDRLNQL	10.6
nsp7	RLQSLQTYV	11.9
nsp8	VVFLHVTYV	17.1
nsp9	NLVPMVATV	17.9
nsp10	GMSRIGMEV	46.4
nsp11	GLCTLVAML	62.2
nsp12	TLDSKTQSL	175.8
nbp1	QVVNVVTTK	28,998.6
nbp2	QLYPEWRTK	18,731.1

Table S3. Synthetic peptides tested with the αβTCRs-transduced Jurkat-TPR system. The nsp peptides were randomly selected among viral peptides predicted to bind the HLA-A*02:01 with an IC_{50} smaller than 500 nM. The predicted binding affinity (IC_{50}) for the peptides and HLA-A*02:01 was computed using NetMHCpan BA 4.0.

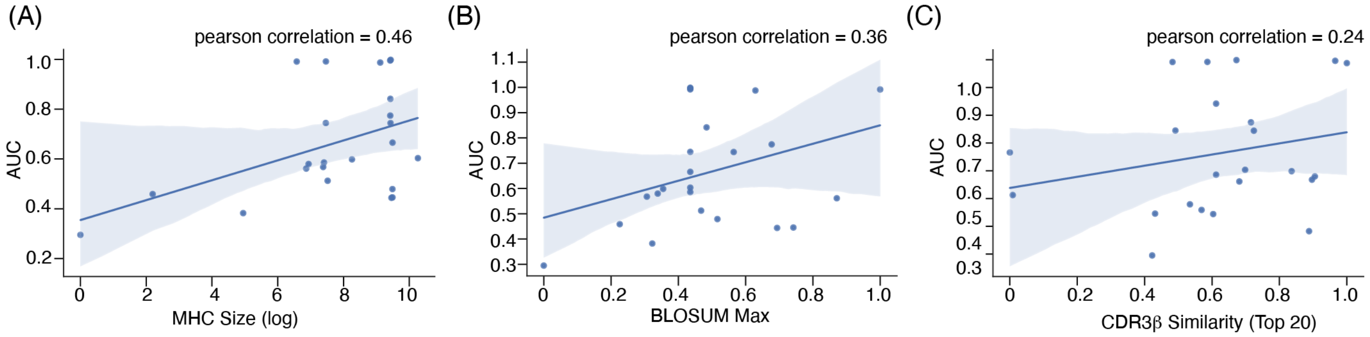

Figure S1. Three properties most moderately correlated with model prediction accuracy. (A-C) Of the properties investigated, these are the ones associated with the highest Pearson correlation with AUC: (A) the log of the number of training examples associated with the same HLA-I allele as that being predicted; (B) the distance between the target peptide and the most similar peptide, as measured using the aligned BLOSUM62 score; and (C) the degree of similarity between the target CDR3β and the most similar CDR3β sequences in the training set, as measured using TCRdist3. None of the features in isolation has a Pearson correlation greater than 0.5.

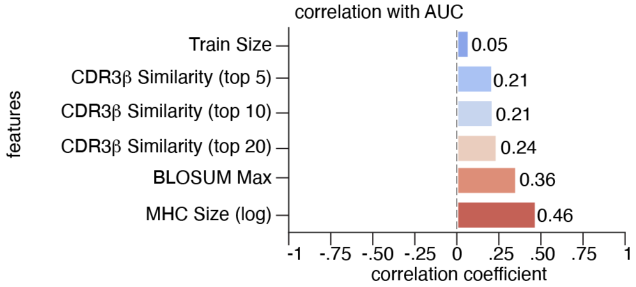

Figure S2. Extended matrix showing the correlation between AUC achieved by the nuTCRacker method and various features relating the training and test sets. The strongest correlation (0.46) is associated with the log of the number of training patterns having the same HLA-I allele as that of the target complex [MHC Size (log)]. The second strongest correlation (0.36) is associated with the similarity (as measured using the BLOSUM62 submission matrix) between the target peptide sequence and the sequence of the most similar peptide in the training set (BLOSUM Max). The third strongest correlation is associated with the similarity (as measured using TCRdist3) between the most similar (though non-identical) CDRH3β sequences in the training set with the sequence of the target CDR3β; here the distance associated with the top 5 most similar sequences - CDR3β Similarity (Top 20) - gives a slightly higher correlation than that associated with the top 10 most similar sequences, *i.e.*, CDR3β Similarity (Top 10) - (Pearson correlation coefficient of 0.24 vs 0.21).

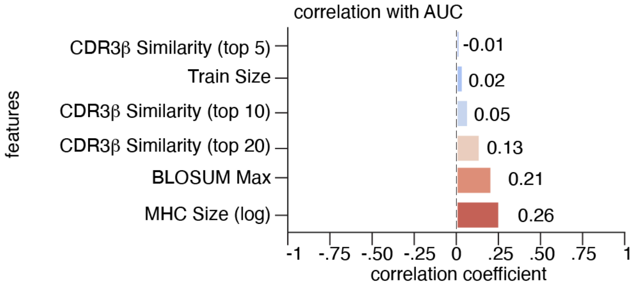

Figure S3. Extended matrix showing the correlation between AUC achieved by the ERGO II method and features relating the training and test sets.

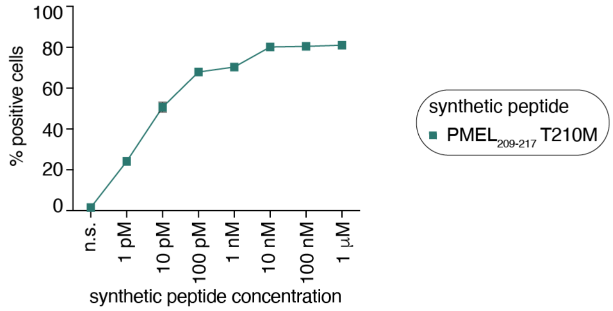

Figure S4. Synthetic peptide concentration dependency of the PMEL$_{209\text{-}217}$-specific αβTCR-transduced Jurkat-TPR activation upon co-culture with K562-A*02:01. Frequency of activated PMEL$_{209\text{-}217}$-specific αβTCR-transduced Jurkat-TPRs upon co-culture with K562-A*02:01 cell line pulsed with different concentration of the synthetic peptide PMEL$_{209\text{-}217}$ T210M [IMDQVPFSV]. The NFAT-eGFP marker was used for measuring the cell activation. Mean and SD (bars) of 2 technical replicates is reported.

References

1 **Springer, I., Tickotsky, N. and Louzoun, Y.,** Contribution of T Cell Receptor Alpha and Beta CDR3, MHC Typing, V and J Genes to Peptide Binding Prediction. *Front Immunol* 2021. **12**: 664514.

2 **Lu, T., Zhang, Z., Zhu, J., Wang, Y., Jiang, P., Xiao, X., Bernatchez, C., Heymach, J. V., Gibbons, D. L., Wang, J., Xu, L., Reuben, A. and Wang, T.,** Deep learning-based prediction of the T cell receptor-antigen binding specificity. *Nat Mach Intell* 2021. **3**: 864-875.

3 **Montemurro, A., Jessen, L. E. and Nielsen, M.,** NetTCR-2.1: Lessons and guidance on how to develop models for TCR specificity predictions. *Front Immunol* 2022. **13**: 1055151.

4 **Jiang, Y., Huo, M. and Cheng Li, S.,** TEINet: a deep learning framework for prediction of TCR-epitope binding specificity. *Brief Bioinform* 2023. **24**.

5 **Mei, S., Li, F., Xiang, D., Ayala, R., Faridi, P., Webb, G. I., Illing, P. T., Rossjohn, J., Akutsu, T., Croft, N. P., Purcell, A. W. and Song, J.,** Anthem: a user customised tool for fast and accurate prediction of binding between peptides and HLA class I molecules. *Brief Bioinform* 2021. **22**.

6 **Cai, M., Bang, S., Zhang, P. and Lee, H.,** ATM-TCR: TCR-Epitope Binding Affinity Prediction Using a Multi-Head Self-Attention Model. *Front Immunol* 2022. **13**: 893247.

7 **Pham, M. N., Nguyen, T. N., Tran, L. S., Nguyen, Q. B., Nguyen, T. H., Pham, T. M. Q., Nguyen, H. N., Giang, H., Phan, M. D. and Nguyen, V.,** epiTCR: a highly sensitive predictor for TCR-peptide binding. *Bioinformatics* 2023. **39**.

8 **Wu, K., Yost, K. E., Daniel, B., Belk, J. A., Xia, Y., Egawa, T., Satpathy, A., Chang, H. Y. and Zou, J.,** TCR-BERT: learning the grammar of T-cell receptors for flexible antigen-xbinding analyses. *bioRxiv* 2021.

9 **Rives, A., Meier, J., Sercu, T., Goyal, S., Lin, Z., Liu, J., Guo, D., Ott, M., Zitnick, C. L., Ma, J. and Fergus, R.,** Biological structure and function emerge from scaling unsupervised learning to 250 million protein sequences. *Proc Natl Acad Sci U S A* 2021. **118**.

10 **Yoo, S., Jeong, M., Seomun, S., Kim, K. and Han, Y.,** Interpretable Prediction of SARS-CoV-2 Epitope-Specific TCR Recognition Using a Pre-Trained Protein Language Model. *IEEE/ACM Transactions on Computational Biology and Bioinformatics* 2024. **21**: 428-438.

11 **Weber, A., Born, J. and Rodriguez Martinez, M.,** TITAN: T-cell receptor specificity prediction with bimodal attention networks. *Bioinformatics* 2021. **37**: i237-i244.

12 **Sidhom, J., Larman, H. B., Pardoll, D. M. and Baras, A. S.,** DeepTCR is a deep learning framework for revealing sequence concepts within T-cell repertoires. *Nature Communications* 2021. **12**: 1605.

13 **Fischer, D. S., Wu, Y., Schubert, B. and Theis, F. J.,** Predicting antigen specificity of single T cells based on TCR CDR3 regions. *Mol Syst Biol* 2020. **16**: e9416.

14 **Moris, P., De Pauw, J., Postovskaya, A., Gielis, S., De Neuter, N., Bittremieux, W., Ogunjimi, B., Laukens, K. and Meysman, P.,** Current challenges for unseen-epitope TCR interaction prediction and a new perspective derived from image classification. *Brief Bioinform* 2021. **22**.

15 **Zhang, W., Hawkins, P. G., He, J., Gupta, N. T., Liu, J., Choonoo, G., Jeong, S. W., Chen, C. R., Dhanik, A., Dillon, M., Deering, R., Macdonald, L. E., Thurston, G. and Atwal, G. S.,** A framework for highly multiplexed dextramer mapping and prediction of T cell receptor sequences to antigen specificity. *Sci Adv* 2021. **7**.

16 **Gielis, S., Moris, P., Bittremieux, W., De Neuter, N., Ogunjimi, B., Laukens, K. and Meysman, P.,** Detection of Enriched T Cell Epitope Specificity in Full T Cell Receptor Sequence Repertoires. *Front Immunol* 2019. **10**: 2820.

17 **Jokinen, E., Huuhtanen, J., Mustjoki, S., Heinonen, M. and Lahdesmaki, H.,** Predicting recognition between T cell receptors and epitopes with TCRGP. *PLoS Comput Biol* 2021. **17**: e1008814.

18 **Fang, Y., Liu, X. and Liu, H.,** Attention-aware contrastive learning for predicting T cell receptor-antigen binding specificity. *bioRxiv* 2022.

19 **Khan, A. R., Reinders, M. J. T. and Khatri, I.,** Determining epitope specificity of T-cell receptors with transformers. *Bioinformatics* 2023. **39**.
